# A Magtein^®^, Magnesium L-Threonate, -Based Formula Improves Brain Cognitive Functions in Healthy Chinese Adults

**DOI:** 10.3390/nu14245235

**Published:** 2022-12-08

**Authors:** Chengxiang Zhang, Qi Hu, Shifen Li, Feifei Dai, Wen Qian, Susan Hewlings, Ting Yan, Yubang Wang

**Affiliations:** 1 The Key Laboratory of Modern Toxicology, Ministry of Education, Center for Global Health, School of Public Health, Nanjing Medical University, Nanjing 210029, China; 2 Safety Assessment and Research Center for Drug, Pesticide and Veterinary Drug of Jiangsu Province, Nanjing Medical University, Nanjing 210029, China; 3 Nutrasoource 120 Research La, Guelph, ON N1G 0B4, Canada

**Keywords:** magnesium L-threonate, magnesium, Magtein^®^, Magtein^®^PS, memory, cognition

## Abstract

Magnesium is one of the most abundant essential minerals in the body. Magnesium supplements mostly have low bioavailability, except magnesium L-threonate. In 2010, a novel magnesium compound, magnesium L-threonate (Magtein^®^) was identified and was shown to raise the magnesium levels in the brain and neurons effectively. In this double-blind, placebo-controlled study, Magtein^®^PS, a magnesium L-threonate (Magtein^®^)- and phosphatidylserine-based formulation additionally containing vitamins C and D, was tested for its cognitive benefits in 109 healthy Chinese adults aged 18–65 years. Subjects were randomly assigned to receive either Magtein^®^PS or placebo (starch) capsules, at a dose of 2 g/day. “The Clinical Memory Test”, the standard test commonly used in Chinese hospitals and academic institutes for cognitive evaluation, was administered before and 30 days after subjects received the supplement. Subjects receiving Magtein^®^PS showed significant improvements over the control group in all five subcategories of “The Clinical Memory Test” as well as the overall memory quotient scores. The older participants showed more improvement than younger participants. Results indicated significant benefits of Magtein^®^PS in improving memory and cognition in healthy Chinese adults.

## 1. Introduction

Magnesium (Mg) is the second-most abundant intracellular mineral, and it is required as a cofactor for over 300 enzymatic reactions and is, thus, necessary for the biochemical functioning of numerous metabolic pathways in the body, including energy generation in every cell, protein production, gene regulation, bone and teeth maintenance, as well as the proper functioning of the brain and nervous systems. Mg is abundant in the food supply and can be found in foods such as grains, cereals and dark leaves, including spinach and cabbage [1]. Despite an abundance in the food supply, intake of Mg in the Chinese population has been shown to be below the 330 mg/day recommended by the China Nutrition Society [2]. For example, the average intake of dietary magnesium was reported to be 205 mg/day, in 2373 adults from Guangxi, China, of both sexes [3]. Therefore, supplementation may be warranted. Mg supplementation has been shown to improve symptoms of migraine headaches, Alzheimer’s disease, stroke and to have a beneficial effect on subjective anxiety in subjects prone to mental stress [4,5]. Higher intake of Mg has been associated with lower depression symptoms [6]. A relationship between Mg and anxiety has also been identified. For example, test anxiety, related to exposure to stressful exam conditions, increases urinary Mg excretion, resulting in a partial reduction in serum Mg levels [7]. A study based on data from the National Health and Nutrition Survey (NHANES) between 2011 and 2014 included 2508 participants aged 60 years and older. After adjusting for multiple confounding variables, they found that total magnesium was independently associated with significantly higher global cognitive scores [8]. Although there is an established need for supplementation, most magnesium compounds available on the market have low bioavailability and do not lead to increased magnesium levels in the brain because they cannot cross the blood–brain barrier easily [9,10]. Thus, it is of great interest to identify a Mg supplement that can elevate brain Mg levels.

In a 2010 publication in the journal *Neuron*, scientists from the Massachusetts Institute of Technology (MIT) reported their discovery of a magnesium compound called magnesium L-threonate (Magtein^®^) that can effectively deliver magnesium to brain cells [11]. According to the study, magnesium L-threonate (Magtein^®^) has greater bioavailability compared to other magnesium supplements. Indeed, when compared to other sources of magnesium, such as chloride, citrate, glycinate and gluconate, magnesium L-threonate (Magtein^®^) demonstrated higher absorption and higher retention [12,13]. In addition, magnesium L-threonate (Magtein^®^) was able to significantly elevate magnesium concentrations (7% to 15% of initial value in 24 days) in cerebrospinal fluid in rats when administered orally, while other magnesium compounds could not [11]. The increased brain levels are most likely due to the increased absorption and the related higher circulating levels of magnesium. In humans, L-threonic acid is an ascorbic acid metabolite [14], having been identified in plasma [15], in the aqueous humor [16], in the urine [17,18] and in the brain [13]. In addition to its endogenous occurrence, L-threonic acid can be found in a wide variety of foods, such as canned mushrooms, fruit juice and processed meats [19] as a major part of vitamin C metabolites.

In a rodent model, Slutsky and colleagues reported that after one month of magnesium L-threonate (Magtein^®^) supplementation, the concentration of magnesium in the brain increased, and there was a significant improvement in memory and learning in both young rats and in elderly rats. In addition, magnesium L-threonate improved memory recovery in elderly rats. Magnesium L-threonate supplementation did not influence body weight, motility or the amount of water and food intake. The possible mechanisms of action of magnesium L-threonate on cognitive functions is via the activation of the NMDA receptors, which leads to increased synaptic density and improved memory [11].

Based on these preclinical results, it would be of great interest to investigate the effects of magnesium L-threonate in a human population. A randomized, double-blind, placebo-controlled study in older American adults (between ages 50 and 70) was published in 2016 [20]; supplementation with magnesium L-threonate significantly improved overall cognitive scores as compared to placebo (*p* = 0.003; Cohen’s d = 0.91). Cognitive fluctuation was also reduced. Aging is associated with magnesium deficiency. A previous study has shown a beneficial effect of magnesium in an older population [21].

Phosphatidylserine (PS) is a natural component of neuronal cell membranes and is required for healthy nerve cell membranes and myelin [22]. Exogenous PS (300–800 mg/day) is absorbed efficiently and crosses the blood–brain barrier and safely slows, halts or reverses biochemical alterations and structural deterioration in nerve cells. PS also supports human cognitive functions, including short-term memory, consolidation of long-term memory, the ability to create new memories, the ability to retrieve memories, the ability to learn and recall information, the ability to focus attention and concentrate, the ability to reason and solve problems, language skills and the ability to communicate [22].

Vitamin B6 deficiency can lead to negative magnesium balance due to increased magnesium excretion [23]. Additionally, vitamin B6 helps facilitate intestinal absorption of magnesium [24]. Therefore, vitamin B6 might have synergistic effects with Mg supplement for treating magnesium deficiency [25].

Vitamin C and D have been added to magnesium L-threonate in a clinical study in patients with mild to moderate dementia, which showed significant improvements in cognition [26]. Vitamin D has been shown to promote Mg reabsorption in the kidney [27], promote Mg absorption in the GI tract [28] and low vitamin D levels have been associated with increased risk of AD [29,30]. Thus, the ingredients in the Magtein^®^PS-based formula could potentially provide synergistic effects by increasing magnesium absorption, increasing brain magnesium levels and increasing cognitive functions. It is the purpose of this study to assess changes in cognitive function with the supplementation of Magtein^®^PS in healthy adults.

## 2. Materials and Methods

Magtein^®^PS, a magnesium L-threonate (Magtein^®^) and phosphatidylserine-based formula was provided by Magceutics, Inc. (Fremont, CA, USA) with the following composition:

Magtein^®^PS (each capsule): 

Magnesium L-threonate (Magtein^®^): 400 mg

Vitamin D3: 80 IU

Vitamin C: 12 mg

Vitamin B6: 4 mg

Phosphatidylserine 50 mg

Magtein^®^PS and placebo (starch) specification: 0.504 g/capsule, recommended dose was 2 g/person/day. Daily dosage was 2 capsules in the morning and 2 capsules in the evening before sleep.

### 2.1. Subject Selection

This study was approved by the Ethics Committee of Safety Assessment and Research Center for Drug, Pesticide and Veterinary Drug of Jiangsu Province (NO. GZ01020150029-5). It was conducted in the Center for Health Safety of Nanjing Medical University and its affiliated hospital located in Hefei, Anhui, China. Subjects were chosen from healthy volunteers (meaning they were free of any diseases as listed below in the exclusion criteria) aged 18 to 65 years old willing to participate by signing the consent form. All subjects were without previous experience participating in similar tests randomized and matched for education level and age. Subjects were screened for basic health parameters by interview and clinical examination. Subjects who reported the following diseases were excluded: heart disease; high blood pressure (≥140/90 mmHg); renal or hepatic impairment/disease; diabetes; bipolar disorder; Parkinson’s disease; Alzheimer’s disease; dementia; thyroid disease; affective disorder or psychiatric disorder diagnosed clinically; immune disorder (such as HIV/AIDS); diagnosed cancer. In addition, the following subjects were excluded: taking drugs containing magnesium such as magnesium sulphate and magnesium chloride; planning a pregnancy or pregnant; values of clinical laboratory examination exceeded normal values.

Comparison analyses were performed in two aspects: before/after within each group, between groups. Experimental group and control group were randomized with age, gender, education balanced. Subjects were randomized based on cognitive function (MQ) using Block randomization. Results of 102 subjects were collected at the end of trial, 51 for Magtein^®^PS group, and 51 for control group. Double blind was achieved by making sure the shape and weight of the placebo and active product were the same in shape and weight and deidentified. One researcher who did not interact with the subject knew the identity of the products.

### 2.2. Testing Methods

The commonly used clinical cognition test, “The Clinical Memory Test” (CMT), published by the Institute of Psychology of the Chinese Academy of Science in 1996, was applied to 109 healthy volunteers. “The Clinical Memory Test” (CMT) is the standard test commonly used in Chinese hospitals and academic institutes for memory and cognitive evaluation. It consists of 5 subtests: directed memory (DM), paired-association learning (PAL), free recall of pictures (FRP), recognition of meaningless figures (RMF), portrait-features memory (PFM) (28). The scaled scores from these five categories of tests, the total score (TS), as well as the memory quotient (MQ) of each participant were recorded at baseline and 30 days after the supplementation.

Directed memory (DM) was assessed by presenting 24 words with a recorder; subjects were then required to memorize12 words from the same category as directed by the recorded guide. One set included a set of fruits and animals, and the other set included a set of vegetables and clothes. Then, there were 12 mixed words which did not need to be remembered but were close to the words that did need to be remembered. For example: The words in the fruit category that were required to be remembered were mixed with various common non-fruit words, such as duck eggs and tofu. The subjects are then asked to recall the mixed words after the second test. This is designed to assess short term memory after hearing words.

Paired-association learning (PAL) was assessed by using 6 pairs of logically connected words and 6 pairs of words without logical connection. Each pairing was tested three times in different order to assess learning, memory and logic.

Free recall of pictures (FRP) is used to assess recall memory. The test is conducted by presenting two groups, each containing 15 stimulating pictures; the pictures are images of daily necessities and other familiar objects, presented in an ordered fashion. Subjects are asked to recall the pictures.

Recognition of meaningless figures (RMF) is used to assess short term memory through vision. The target stimuli are five types of meaningless graphics, curve closed, straight line closed, curve straight line, curves not closed and straight lines not closed. Training starts with four figures from targeted stimuli group, presentation 1 s flowed by an interval of 1 s and presented in sequence; then, mixed 20 pictures of target stimuli group with 20 pictures of other groups, presented in a random order for 3 s followed by an interval of 3 s, requiring the participant to recognize the targeted stimuli group figures. 

Portrait-features memory (PFM) is designed to test for more complicated memory involving several parts of the brain. Six face sketch portraits, each showing for 9 s, with an interval of 3 s, showing the “surname”, “career” and “hobby” of the portrait at the same time (such as surname Wu, actor, loves swimming), presented in sequence and repeated once. Then, presenting in another order, we asked the participant to say the last name, career and hobby when presenting each portrait.

Memory quotient (MQ) is a cognition score adjusted for age and education. CMT test is the standard test used in hospitals and research institutions in China for memory and cognition abilities.

Measurements from these 5 categories can be converted into scale points. The sum of the scaled scores from these five categories is the total score (TS) of the participant. From the table of the published CMT book, based on the participant’s age and educational status, memory quotient (MQ) can be found corresponding to TS of this individual.

### 2.3. Data Analysis

Parallel comparisons between groups were analyzed by using *t*-test of two samples’ mean. Self-reference data were analyzed by using paired *t*-test. When variance was uneven, data conversion was conducted by using *t*-test and rank sum test. Effective rate and total effective rate were calculated via *x*^2^ test. In the case that the total number of cases in the four-square table was less than 40, or the total number of cases was equal to or greater than 40, but the theoretical number was equal to or less than 1, the exact probability method was used instead.

### 2.4. Instruments

The following instruments were used to evaluate general health of the participants at baseline. Automatic biochemical analyzer (A25Biosysems); automatic hematology analyzer (BC-3000 Plus); automatic urine analyzer (Uritest-300); B-ultrasound set (RH-3200); X-ray machine (Imax-1500z); ECG machine (AIKD-B-12).

### 2.5. Safety Parameters

The following tests were measured for all participants as safety parameters: General health indicators: mental condition, sleep, diet, excretion, heart rate, blood pressure; Blood/urine test: blood test includes white blood cells, red blood cells, hemoglobin and platelets; Blood biochemical test: checking list includes serum total protein, albumin, alanine, aminotransferase, aspartate aminotransferase, urea, creatinine, cholesterol, triglycerides, blood glucose; Chest X-rays, ECG, B-ultrasound tests: only performed once at the beginning of the study.

## 3. Results

There were a total 109 participants enrolled in the study, including 54 subjects for the experimental group and 55 subjects for the control group. A total of 102 subjects finished this trial, 51 subjects for the experimental group and 51 subjects for the control group. Three subjects in experimental group and four subjects in control group failed to follow up due to loss of contact; they did not provide any reason for their lack of participation. The total drop-out rate was 6.42%.

### 3.1. Subject Characteristics

Data from 102 subjects were collected, which includes the experimental group consisting of 51 subjects, 24 male and 27 female, average age 41.04 years, and the control group consisting of 51 subjects, 19 male and 32 female, average age 42.47 years. As seen in Table 1, the differences of memory quotient (MQ), age, gender, education level between experimental group and control group was not statistically significant (*p* > 0.05).

It indicates that the two groups are comparable in MQ, age, gender and education level at the beginning of the study. No abnormality was observed from chest X-rays, ECG or abdominal B-ultrasound tests from subjects in either group.

### 3.2. Safety Parameters between Experimental Group and Control Group

To assess the impact of Magtein®PS on recognized safety endpoints, mental condition, sleep, diet and bowel habits were monitored throughout the study and categorized as good, normal. Blood pressure and heart rate were also tested and are shown in Table 2. There were no significant differences in these parameters between those in the experimental group compared to those in the placebo group, indicating that Magtein^®^PS had no negative effect on the parameters evaluated.

As shown in Table 3, the blood biochemical levels of participants in both groups were in the normal range before and after the study. Urine tests from both groups were also normal before and after the study. Therefore, Magtein^®^PS had no negative effects on blood or urine biochemical parameters.

Thus, within the parameters assessed in this study it can be concluded that Magtein^®^PS is safe for humans, at least in this study.

### 3.3. Cognitive Parameter between Experimental Group and Control Group

#### 3.3.1. Between-Group Analysis

As seen in Table 4, after 30 days, the average score for directed memory (DM), paired-association learning (PAL), free recall of pictures (FRP), recognition of meaningless figures (RMF), portrait-features memory (PFM) and memory quotient (MQ) in the Magtein^®^PS group was significantly higher compared to the placebo group (*p* < 0.001).

#### 3.3.2. Within Group Analysis

**Figure 1:**
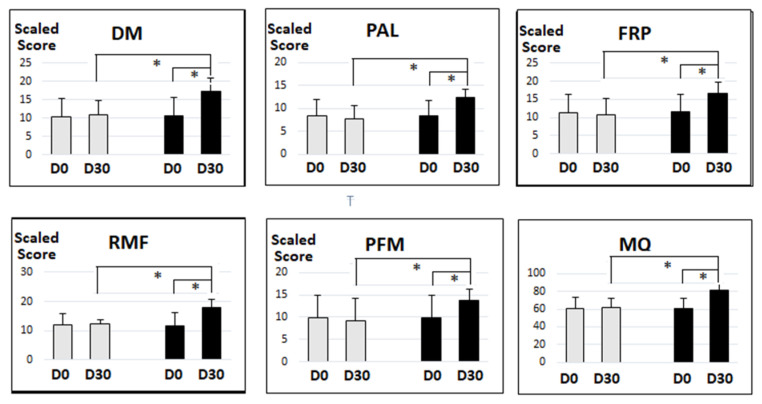
The significant impact of Magtein^®^PS on cognition. *: *p* value (*p* < 0.001), Grey box: placebo; black box: Magtein^®^PS. D0 = Day 0, D30 = Day 30.

Within study comparison revealed that the average score of the Magtein^®^PS group after the trial was significantly higher than before the trial for all clinical memory tests (*p* < 0.001). There was no significant improvement of the average score for any clinical memory test in the placebo group before and after the trial (*p* > 0.05). Moreover, for PFM, the score was significantly worse after the trial when compared to before the trial in the placebo group (*p* = 0.017). Figure 1 summarizes the overall results on all clinical memory tests, including the 5 subcategories as well as the overall MQ scores.

### 3.4. Benefit of Magtein^®^PS on the Cognitive Function Increases with Age

**Figure 2:**
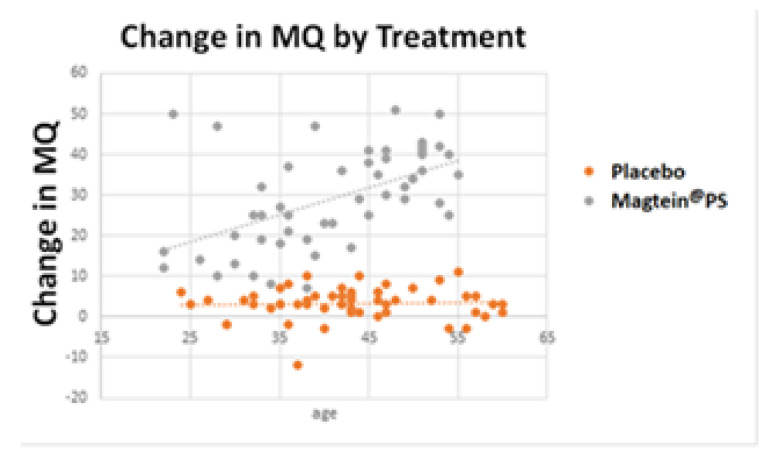
The effect of Magtein^®^PS on MQ increases with age *p* value (*p* < 0.001).

A further analysis was conducted to assess any potential age-related differences in this study. As seen in Figure 2, Magtein^®^PS supplementation led to improved MQ in all age groups, and the impact is positively associated with age (*p* < 0.001): the older the participants, the higher the improvement from Magtein^®^PS intake.

## 4. Discussion

The benefits associated with the individual ingredients of Magtein^®^PS, a magnesium L-threonate (Magtein^®^) and phosphatidylserine-based formulation additionally containing vitamins C and D suggest a plausible synergistic benefit. Therefore, in this study, we tested a formula containing the above listed ingredients. We found that the group receiving Magtein^®^PS demonstrated significant improvements (*p* < 0.001) in all categories of the cognition tests measured (Table 4, Figure 1 and Figure 2), consistent with our synergistic hypothesis. Interestingly, the older participants experienced the highest improvement in cognition (Figure 2).

A previous publication of a double-blind, placebo-controlled human clinical examining the effects of Magtein^®^ in older participants demonstrated significant elevation of brain Mg levels as well as cognitive abilities in the supplemented group as compared to the placebo [20]. It is interesting to note that the effective elemental magnesium levels used for the observed benefits of magnesium L-threonate (Magtein^®^) were 108–144 mg/day which is below the RDA of 350–420 mg/day. Thus, supplementation even when combined with dietary intake would likely be within safe limits.

Even though, in this study, we only assessed cognitive function, it is reasonable that the benefits of Magtein^®^ may provide benefits for multiple applications. For example, it has been suggested that magnesium supplementation may be ideal for those with treatment-resistant depression [31]. Results as to the connection of magnesium intake, magnesium levels and depression are mixed. This is thought to be due to methodology in that most studies do not measure brain magnesium levels. Brain levels of magnesium are found to be lower in in patients with depression as well as other forms of mood disturbances [31], suggesting that a magnesium supplement that could readily cross the blood–brain barrier would be beneficial over those that do not readily cross the blood–brain barrier. Increasing brain levels of magnesium would provide potential for many psychological and neurological conditions such as migraine headaches, Alzheimer’s disease, stroke and subjective anxiety in subjects prone to mental stress. Perhaps this explains the benefits experienced with magnesium supplementation in subjects with these conditions [4,5]. 

The potential for a benefit is supported by evidence related to a mechanism of action indicating that magnesium plays a key role in the regulation of N-methyl-D-aspartate (NMDA) receptor excitability in the brain. NMDA causes degeneration of neurons and magnesium intake supports healthy neurons by blocking the activity of NMDA. Blocking NMDA is thought to be a potential prevention for cognitive impairment and Alzheimer’s disease [32]. Additionally, magnesium deficiency has been shown to increase inflammatory mediators leading to neuroinflammation which is said to enhance progression of cognitive impairment and dementia [33]. A recent meta-analysis of 17 randomized controlled trials showed that Mg supplementation significantly decreased serum C-reactive protein (CRP) and increased nitric oxide (NO) levels, an important vasodilator [34]. In the brain, NO enhances blood flow and has a key role in intracellular signaling in neurons [35] The proposed mechanisms of action combined with epidemiological data showing a relationship between total magnesium and significantly higher global cognitive scores [8] support the findings of our study. The finding that the older subjects demonstrated the greatest improvement could be explained by lower brain levels of magnesium or perhaps a great degeneration of the neurons responding more notably to the increased magnesium.

The limitations of this study include a small sample size, just 51 people in the treatment group contributing to a small number of people in each age group when sub-analyses were conducted. Additionally, brain levels of magnesium were not assessed in this study, which may have been interesting to determine. Serum magnesium levels were not measured in this study as the value was not central to the purpose of this study. Further, it has been suggested that serum levels may not be the best indicator of brain levels; it may be interesting to measure this in future studies. This was not a diverse population as all study subjects were Chinese. Though subjects were instructed not to change their normal diet, we cannot entirely control for potential changes in dietary intake of magnesium during the study time period. Future studies could consider a larger, more diverse subject population. Sub-analysis for age, gender and other factors known to correlate with magnesium levels may also be interesting to evaluate. Despite a few limitations, our study was well powered and cognitive function was evaluated using a well-known and validated assessment tool.

## 5. Conclusions

This double-blind, placebo-controlled study demonstrates that Magtein^®^PS is well tolerated and safe within the parameters of this study. Our data supported the benefits of Magtein^®^PS on improving learning, recall, memory and cognitive abilities in this group of healthy Chinese adults. Furthermore, the benefits of Magtein^®^PS were observed among all ages, with older people demonstrating the most improvement. These findings may support cognitive function as well as other benefits in all age groups, especially older adults.

## Figures and Tables

**Table 1:** Baseline distribution comparison of two groups before clinical trial.

Index	Experimental Group	Control Group	*p* *
MQ	60.31 ± 11.35	60.75 ± 12.31	0.854
Age (years)	41.04 ± 9.41	42.47 ± 9.40	0.444
Male/Female	24/27	19/32	0.423

* *p* represents the comparison between experimental group and control group.

**Table 2:** General comparison between two groups before and after clinical trial.

	Experimental Group							Control Group						
	Before Clinical Trial			After Clinical Trial				Before Clinical Trial			After Clinical Trial			
	Good	Normal	Bad	Good	Normal	Bad	*p* ^1^	Good	Normal	Bad	Good	Normal	Bad	*p* ^1^
Mental condition	50	1	0	51	0	0	1.000	51	0	0	51	0	0	1.000
Sleep condition	50	1	0	51	0	0	1.000	51	0	0	51	0	0	1.000
Appetite	50	1	0	51	0	0	1.000	51	0	0	51	0	0	1.000
Exercise	50	1	0	51	0	0	1.000	51	0	0	51	0	0	1.000
Systolic blood pressure (mmHg)	120.00 ± 8.60			119.31 ± 7.36			0.184	119.65 ± 7.40			119.65 ± 7.40			0.124
Diastolic blood pressure (mmHg)	76.24 ± 3.94			76.25 ± 3.82			0.957	76.55 ± 3.60			76.55 ± 3.60			0.203
Heart rate	69.39 ± 9.69			70.37 ± 7.56			0.070	71.20 ± 9.87			70.82 ± 9.87			0.583

*p*^1^ represents the self-comparison before and after clinical trial.

**Table 3:** The effect of Magtein^®^PS on levels of human blood and urine biochemical parameters.

Index	Before Clinical Trial			After Clinical Trial		
	Experimental Group	Control Group	*p*	Experimental Group	Control Group	*p*
Leukocyte (10^9^/L)	6.15 ± 1.73	5.90 ± 1.17	0.389	6.25 ± 1.42	5.87 ± 1.46	0.180
RBC (10^12^/L)	4.56 ± 0.6	4.38 ± 0.44	0.084	4.62 ± 0.53	4.46 ± 0.46	0.110
Platelet (10^9^/L)	176.2 ± 48.65	176.2 ± 48.21	1.000	188.61 ± 50.6	200.22 ± 46.34	0.230
Hemoglobin (g/L)	136.27 ± 17.79	130.9 ± 17.93	0.132	135.94 ± 16.57	130.59 ± 19.36	0.137
Total protein (g/L)	71.70 ± 3.82	71.05 ± 3.09	0.352	74.54 ± 5.38	75.35 ± 3.78	0.384
Albumin (U/L)	46.38 ± 2.46	45.39 ± 2.29	0.067	48.15 ± 4.67	48.51 ± 2.53	0.627
Alanine Aminotransferase (U/L)	20.68 ± 15.00	23.7 ± 25.11	0.464	20.1 ± 12.92	24.00 ± 27.38	0.359
Aspartate transaminase (U/L)	19.39 ± 5.61	20.2 ± 12.23	0.671	19.04 ± 4.97	20.88 ± 12.66	0.335
Urea (mmol/L)	5.6 ± 1.6	5.31 ± 1.32	0.325	5.03 ± 1.36	4.90 ± 1.38	0.624
Creatinine (umol/L)	62.73 ± 11.16	59.61 ± 14.62	0.229	72.78 ± 13.80	68.8 ± 15.9	0.180
Blood sugar (mmol/L)	5.68 ± 0.5	6.08 ± 1.68	0.101	4.72 ± 0.57	5.17 ± 1.23	0.071
Cholesterol (mmol/L)	5.37 ± 0.69	5.18 ± 0.88	0.213	4.67 ± 0.85	4.53 ± 0.88	0.408
Triglyceride (mmol/L)	1.55 ± 1.83	1.31 ± 0.71	0.385	1.61 ± 1.45	1.63 ± 1.85	0.953
Urine test	Normal	Normal		Normal	Normal	

*p* represents the self-comparison before and after clinical trial.

**Table 4:** Effects of Magtein PS and placebo on CMT Scores and MQ score by treatment.

CMT Item	Period	Magtein^®^ PS		Placebo		*p*-Value ^b^
		Mcan ± SD	*p*-Value ^a^	Mcan ± SD	*p*-Value ^a^	
DM	Day 0	10.69 ± 4.98	<0.001	10.47 ± 5.00	0.222	0.828
	Day 30	17.20 ± 4.26		10.98 ± 3.94		<0.001
PAL	Day 0	8.37 ± 3.26	<0.001	8.27 ± 3.56	0.263	0.885
	Day 30	12.37 ± 2.61		7.7 ± 2.95		<0.001
FRP	Day 0	11.47 ± 5.04	<0.001	11.39 ± 5.11	0.073	0.938
	Day 30	16.65 ± 3.07		10.75 ± 4.46		<0.001
RMF	Day 0	11.73 ± 4.61	<0.001	12.16 ± 3.54	0.840	0.597
	Day 30	17.88 ± 2.73		12.24 ± 1.54		<0.001
PFM	Day 0	9.82 ± 5.08	<0.001	9.76 ± 5.11	0.017	0.954
	Day 30	13.75 ± 2.42		9.20 ± 4.97		<0.001
MQ	Day 0	60.31 ± 11.35	<0.001	60.75 ± 12.31	0.206	0.854
	Day 30	81.84 ± 7.18		61.73 ± 10.27		<0.001

Notes: ^a^ *p*-value comparison within the two study groups, ^b^ *p*-value comparison of changes from baseline between the two study groups. Abbreviations: CMT: Clinical Memory Test; DM: directed memory; PAL: paired-association learning; FRP: free recall of pictures; RMF: recognition of meaningless figures; PFM: portrait features memory; MQ: memory quotient.
